# Virulence of the Melioidosis Pathogen Burkholderia pseudomallei Requires the Oxidoreductase Membrane Protein DsbB

**DOI:** 10.1128/IAI.00938-17

**Published:** 2018-04-23

**Authors:** Róisín M. McMahon, Philip M. Ireland, Derek S. Sarovich, Guillaume Petit, Christopher H. Jenkins, Mitali Sarkar-Tyson, Bart J. Currie, Jennifer L. Martin

**Affiliations:** a Institute for Molecular Bioscience, The University of Queensland, St. Lucia, Queensland, Australia; b Griffith Institute for Drug Discovery, Griffith University, Nathan, Queensland, Australia; c Defence Science and Technology Laboratory, Chemical, Biological and Radiological Division, Porton Down, Salisbury, Wiltshire, United Kingdom; d Global and Tropical Health Division, Menzies School of Health Research, Darwin, Northern Territory, Australia; e Faculty of Science, Health, Education and Engineering, University of the Sunshine Coast, Sippy Downs, Queensland, Australia; f Marshall Centre for Infectious Disease Research and Training, School of Pathology and Laboratory Medicine, University of Western Australia, Perth, Western Australia, Australia; University of Michigan—Ann Arbor

**Keywords:** Burkholderia pseudomallei, disulfide bond protein, melioidosis, X-ray crystallography, oxidoreductases, virulence determinants

## Abstract

The naturally antibiotic-resistant bacterium Burkholderia pseudomallei is the causative agent of melioidosis, a disease with stubbornly high mortality and a complex, protracted treatment regimen. The worldwide incidence of melioidosis is likely grossly underreported, though it is known to be highly endemic in northern Australia and Southeast Asia. Bacterial disulfide bond (DSB) proteins catalyze the oxidative folding and isomerization of disulfide bonds in substrate proteins. In the present study, we demonstrate that B. pseudomallei membrane protein disulfide bond protein B (BpsDsbB) forms a functional redox relay with the previously characterized virulence mediator B. pseudomallei disulfide bond protein A (BpsDsbA). Genomic analysis of diverse B. pseudomallei clinical isolates demonstrated that *dsbB* is a highly conserved core gene. Critically, we show that DsbB is required for virulence in B. pseudomallei. A panel of B. pseudomallei *dsbB* deletion strains (K96243, 576, MSHR2511, MSHR0305b, and MSHR5858) were phenotypically diverse according to the results of *in vitro* assays that assess hallmarks of virulence. Irrespective of their *in vitro* virulence phenotypes, two deletion strains were attenuated in a BALB/c mouse model of infection. A crystal structure of a DsbB-derived peptide complexed with BpsDsbA provides the first molecular characterization of their interaction. This work contributes to our broader understanding of DSB redox biology and will support the design of antimicrobial drugs active against this important family of bacterial virulence targets.

## INTRODUCTION

Melioidosis is a tropical disease caused by the soil-dwelling bacterium and tier 1 select agent Burkholderia pseudomallei. The clinical presentations of melioidosis are myriad; however, the disease is classically characterized by pneumonia with or without sepsis. The disease can vary greatly in severity from a mild, self-resolving skin abscess to rapidly fatal, acute pneumonia with septic shock (1). Mortality rates are high and range from 10 to 40%, depending on the geographical region (2).

In tropical northern Australia, the bacterium is considered endemic (3) and is a leading cause of fatal pneumonia and sepsis (3, 4). Recent global modeling of disease based on environmental prevalence and confirmed cases in animals and humans suggests that the global prevalence of B. pseudomallei and the incidence of melioidosis are likely much greater than previously thought, with an estimated 165,000 (95% credible interval, 68,000 to 412,000) human melioidosis cases per year worldwide, of which 89,000 (95% credible interval, 36,000 to 227,000) are fatal (5). In Australia, there has been a recent sharp increase in the infection rate. Between 2009 and 2012 there were 252 cases, in comparison to 540 cases in the previous 2 decades (6). This was attributed in part to the local emergence of a novel strain of apparent Asian origin (7) and increased heavy rainfall during the period and, thus, elevated exposure to the bacteria (6). Analysis of the association of melioidosis with climatic factors in Darwin, Australia, over a 23-year period identified a statistical association between the frequency of recorded cases and the nature and timing of rainfall-related events and suggests that a future rise in the sea surface level and the ambient temperature may lead to an increased incidence of melioidosis (8). In addition, anthropogenic environmental change across northern Australia, such as the importation of pasture grasses that appear to be hosts for the propagation of B. pseudomallei, may alter the soil ecology in favor of B. pseudomallei (9).

Despite significant investment in research to identify a melioidosis vaccine, there are none on the horizon. A number of vaccine candidates have been demonstrated to provide partial protection in murine models of infection, but none has yet achieved sterilizing immunity or progressed to nonhuman primate or human trials (10). Treatment relies on prolonged intravenous/oral antibiotic regimes that can last up to 6 months (11, 12).

The bacterial disulfide bond (DSB) machinery catalyzes the oxidation and isomerization of disulfides in secreted and membrane proteins and is a pivotal control point for bacterial virulence. The oxidative folding machinery in Gram-negative bacteria typically comprises a soluble periplasmic oxidase (disulfide bond protein A [DsbA]) and its partner membrane protein (disulfide bond protein B [DsbB]) (13). Together with a quinone cofactor, DsbB generates *de novo* disulfide bonds which it donates to DsbA, which in turn oxidizes unfolded or partially folded substrate proteins (14). There is now overwhelming evidence for the role of DSB proteins in the pathogenicity of bacteria, including Vibrio cholerae (15), Escherichia coli (16), Francisella tularensis (17), and others reviewed in reference 18.

In B. pseudomallei, deletion of *dsbA* has marked pleiotropic effects on virulence (19). B. pseudomallei strains deficient in *dsbA* show altered secretion of protease and a reduced capacity to replicate in macrophages and are less motile than their wild-type (WT) counterparts. Strikingly, in a mouse model of infection, *dsbA* is required for virulence; while all mice infected with WT bacteria die within 42 days, those infected with Δ*dsbA* bacteria survive. This differential outcome indicates the pivotal role of DSB proteins in disease.

In the present study, we demonstrate that B. pseudomallei membrane protein DsbB (BpsDsbB) is the redox partner of B. pseudomallei DsbA (BpsDsbA) and is highly conserved across a diverse range of isolates. B. pseudomallei with the *dsbB* deletion is attenuated in a mouse model of infection, likely the consequence of disruption of BpsDsbA-mediated virulence. We also report a 2.5-Å resolution crystal structure of BpsDsbA in complex with a peptide from the predicted interaction loop of BpsDsbB. This structure provides the first molecular snapshot of how BpsDsbB may engage with BpsDsbA. Comparison with E. coli DsbA (EcDsbA) in complex with a peptide derived from its partner protein, E. coli DsbB, reveals commonalties of binding. This knowledge supports ongoing efforts to develop inhibitors of both classes of enzyme.

## RESULTS

### DsbA and DsbB are highly conserved among a diverse collection of B. pseudomallei clinical isolates.

**FIG 1:**
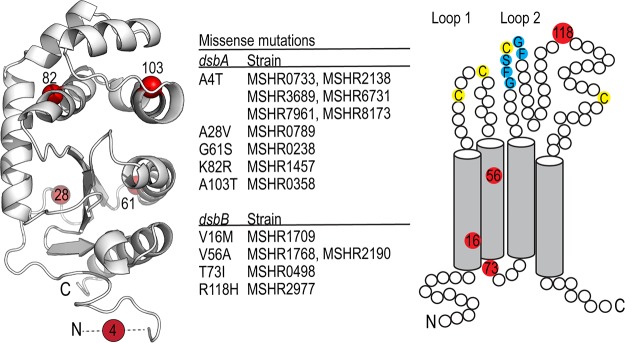
DsbA and DsbB are highly conserved among a diverse collection of B. pseudomallei isolates. Analysis of the genetic variation of DsbA and DsbB among 431 clinical isolates of B. pseudomallei sourced from the Darwin Prospective Melioidosis Study found both genes to be highly conserved. The vast majority of isolates possess a sequence identical to that of strain K96243, with only a few mutations being observed in a limited number of strains (center). The observed five missense mutations (Ala4Thr, Ala28Val, Gly61Ser, Lys82Arg, and Ala103Thr) observed for BpsDsbA are mapped onto the crystal structure of BpsDsbA (PDB accession number 4K2D, 1.9-Å resolution) (left). Mutation positions are colored red, and the relevant C-α atom is shown as a sphere. The 6 most N-terminal residues could not be resolved in the electron density of this crystal structure and are represented by a dashed line. For BpsDsbB, we observed four missense mutations (Val16Met, Val56Ala, Thr73Ile, and Arg118His), again in only a few of the 431 isolates (center). The positions of the mutations (red circles) are depicted in a schematic of BpsDsbB. Transmembrane helix boundaries were predicted using the TMHMM server (76, 77). Key cysteine residues in each periplasmic loop and the predicted BpsDsbA interaction sequence Gly-Phe-Ser-Cys-Gly-Phe (Fig. 5) are marked in blue.

Bioinformatic analyses predicted that B. pseudomallei encodes a homolog of E. coli DsbB (EcDsbB) and a likely redox partner of BpsDsbA. We analyzed the genetic variation of DsbA and DsbB among 431 clinical isolates of B. pseudomallei sourced from the Darwin Prospective Melioidosis Study, which has documented melioidosis cases occurring in northern Australia over the past 25 years (20). Both *dsbB* and *dsbA* are contained in the core genome of B. pseudomallei, with all isolates analyzed containing complete sequences for these genes. Both *dsbA* and *dsbB* are highly conserved, with the vast majority of isolates possessing sequences identical to strain K96243. For BpsDsbA, we observed five missense mutations (Ala4Thr, Ala28Val, Gly61Ser, Lys82Arg, and Ala103Thr [K96243 annotation with a 15-amino-acid signal sequence removed]) in only a few isolates (Fig. 1). For BpsDsbB, we observed four missense mutations (Val16Met, Val56Ala, Thr73Ile, and Arg118His) in only a few isolates; Val16Met, Thr73Ile, and Arg118His were found in single isolates, and Val56Ala was identified in two isolates (Fig. 1). No correlation with disease severity or clinical presentation was observed for any of these variants (data not shown).

### BpsDsbA and BpsDsbB are redox partners.

**FIG 2:**
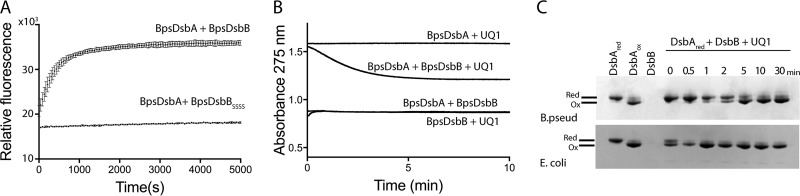
BpsDsbA and BpsDsbB are redox partners. (A) BpsDsbB sustains BpsDsbA-catalyzed folding of a model peptide substrate. BpsDsbA (80 nM) and BpsDsbB (1.6 μM) (crude membrane preparation) efficiently catalyze the oxidation of a peptide substrate, as indicated by monitoring an oxidation-dependent fluorescence signal. Means and SDs are shown (*n* = 4). BpsDsbB in which each periplasmic cysteine residue has been mutated to serine (BpsDsbB_SSSS_) is unable to sustain BpsDsbA oxidative catalytic activity. Means and SDs are shown (*n* = 2). (B) Reduced BpsDsbA catalyzes BpsDsbB-mediated reduction of ubiquinone, as monitored by a decrease in ubiquinone absorption at 275 nm. Reaction mixtures lacking BpsDsbB, BpsDsbA, or UQ1 exhibited no *A*_275_ change. (Note that the difference in the starting absorbance arose from the different intrinsic absorptions of BpsDsbA, BpsDsbB, and mixes thereof.) The data presented are representative of those from three separate experiments. (C) Reduced (Red) BpsDsbA is converted to oxidized (Ox) BpsDsbA by a catalytic amount of detergent-solubilized purified BpsDsbB in the presence of UQ1. The reaction mixture was sampled over 30 min, and the reaction was stopped with TCA precipitation. Following AMS alkylation of free thiols (which adds 0.5 kDa to each cysteine in the reduced enzyme), reduced and oxidized BpsDsbA were separated on the basis of their different electrophoretic mobilities. Control reactions used E. coli DsbA and E. coli DsbB. The experiments were repeated on three independent occasions, and representative data from one of those experiments are reported.

To test the hypothesis that BpsDsbB is the redox partner of BpsDsbA, we recombinantly expressed BpsDsbB in E. coli. Membranes containing BpsDsbB can sustain the BpsDsbA-catalyzed folding of a model peptide substrate (Fig. 2A) in a manner similar to that for the small oxidizing molecule oxidized glutathione (19), but membranes containing a variant of BpsDsbB in which each of the periplasmic cysteine residues has been mutated to serine (BpsDsbB_SSSS_) cannot. This suggests that BpsDsbA is a specific redox partner of BpsDsbA (Fig. 2A). Consistent with this, purified detergent-solubilized BpsDsbB can reduce ubiquinone in the presence of reduced BpsDsbA. In the canonical E. coli DSB system, a ubiquinone cofactor accepts electrons from EcDsbB to enable EcDsbB-mediated oxidation of reduced EcDsbA (14). Ubiquinone-1 (UQ1) is also reduced (as followed spectrophotometrically by a decrease in the absorbance at 275 nm) when mixed with reduced BpsDsbA and BpsDsbB (Fig. 2B). By comparison, reactions in which BpsDsbA, BpsDsbB, or UQ1 was omitted did not exhibit any change in the *A*_275_ spectrum over time. Additionally, a catalytic amount of purified detergent-solubilized BpsDsbB could convert purified reduced BpsDsbA to its oxidized form in the presence of UQ1 (Fig. 2C). After BpsDsbA was mixed with BpsDsbB and UQ1, the redox state of BpsDsbA was assessed over time on the basis of the differential electrophoretic mobility of its reduced and oxidized forms. Within 5 min, reduced BpsDsbA was fully converted to oxidized BpsDsbA (Fig. 2C). Control reactions using E. coli proteins showed the same progression, though with a more rapid conversion to its oxidized form. Taken together, these data demonstrate that BpsDsbA and BpsDsbB are redox partners.

### *In vitro* growth of B. pseudomallei is not affected by *dsbB* deletion.

To investigate the potential impact of BpsDsbB on bacterial replication, *in vitro* growth curves were produced for selected strains. No difference in aerobic growth was observed between WT B. pseudomallei and the Δ*dsbB* mutant of strain K96243 or MSHR2511 (see Fig. S1A and B in the supplemental material). The growth of B. pseudomallei in the absence of oxygen was monitored in medium containing 25 mM sodium nitrate (21). The growth of WT strain K96243 and the K96243 Δ*dsbB* mutant under anaerobic conditions did not differ over the time course (Fig. S1C). The growth of WT B. pseudomallei in medium without sodium nitrate (optical density at 590 nm [OD_590_], 0.19 by 12 h) was minimal and as previously reported was presumably a consequence of residual dissolved oxygen in the culture medium (21).

### *dsbB* is important for protease secretion and motility in some B. pseudomallei strains.

**FIG 3:**
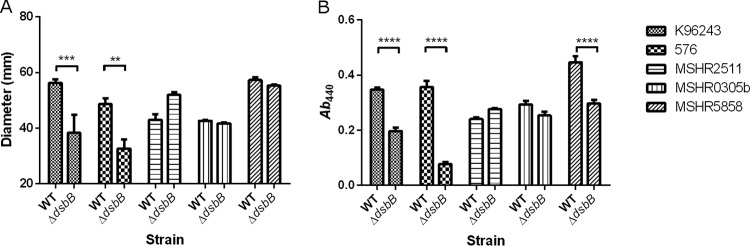
Deletion of *dsbB* affects motility (A) and protease secretion (B) in some B. pseudomallei strains. Mean values from three biological replicates are plotted with the standard error of the mean. A two-way ANOVA was followed by Sidak's multiple-comparison test. **, *P* < 0.01; ***, *P* < 0.001; ****, *P* < 0.0001.

To study the impact of *dsbB* deletion on B. pseudomallei physiology, phenotypes typically associated with disruption to the DSB system were examined across a number of B. pseudomallei strains. Strains K96243 Δ*dsbB* and 576 Δ*dsbB* were significantly less motile in 0.3% LB agar than the WT, with the mean zone of motility being reduced from 56 mm to 38 mm and from 49 mm to 33 mm, respectively (Fig. 3A). The less severe reduction in motility observed for these *dsbB* deletion strains than for a *dsbA* deletion strain (19) is consistent with motility data reported previously (22, 23). In contrast, no difference in motility was observed between WT parental strains and their Δ*dsbB* variants in the Australian clinical isolates MSHR2511, MSHR0305b, and MSHR5858. Further motility studies using 0.3% Muller-Hinton agar and a 0.3% defined medium also showed no difference in motility between the WT and Δ*dsbB* mutant of the Australian clinical isolates.

Compounds such as cystine have previously been shown to chemically complement the *dsbB* mutation in other bacteria (22, 23). Therefore, the minimal medium M9 was selected for use for the preparation of a B. pseudomallei culture supernatant. Protease activity was evident in culture supernatants prepared from all strains tested (Fig. 3B). Three of the *dsbB* deletion strains, including K96243, 576, and MSHR5858, exhibited a significant reduction in protease activity, resulting in 1.76-, 4.65-, and 1.51-fold reductions in activity, respectively, compared to that of the WT (Fig. 3B). Trypsin (28.6 units) was used as a positive control for azocasein hydrolysis, reaching a mean *A*_440_ value of 0.33 ± 0.009 (standard error of the mean [SEM], *n* = 3). Heat-treated culture supernatant (80°C for 20 min) served as a negative control for each sample, with mean *A*_440_ values ranging from 0.01 ± 0.003 to 0.02 ± 0.009 (SEM). The effect of the *dsbB* deletion in B. pseudomallei strains grown in LB broth followed the same trend as the effect of the deletion in B. pseudomallei strains grown in M9 medium.

### B. pseudomallei Δ*dsbB* strains are highly attenuated in a murine model of melioidosis.

To study the role of *dsbB* in the virulence of B. pseudomallei, two strains were selected for study within a murine model of infection. These strains were K96243 Δ*dsbB*, which had exhibited altered phenotypes under the *in vitro* conditions tested, and MSHR2511 Δ*dsbB*, which exhibited phenotypes comparable to those of the WT.

**FIG 4:**
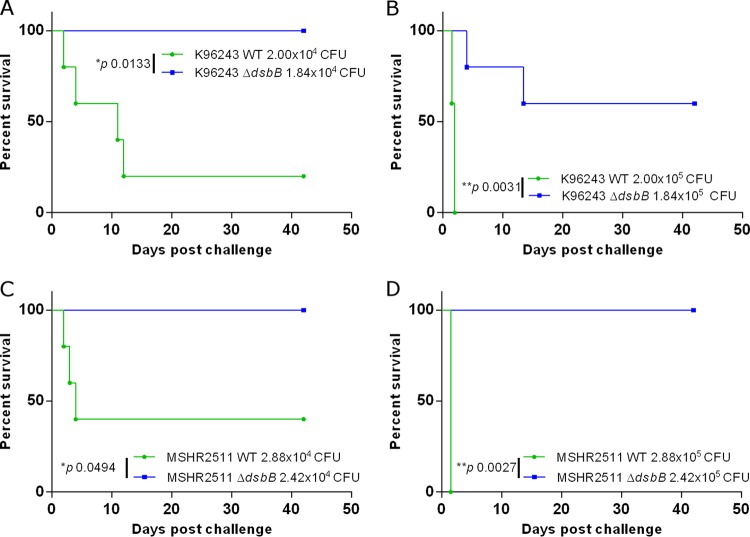
B. pseudomallei Δ*dsbB* is significantly attenuated in a murine model of infection. Groups of 5 mice were challenged via the i.p. route of infection with B. pseudomallei. The impact of *dsbB* deletion on virulence was assessed for strains K96243 (A and B) and MSHR2511 (C and D). Statistically significant differences were determined by the log rank (Mantel-Cox) test.

When mice were challenged with 2 × 10^4^ CFU WT K96243, 80% of mice succumbed to the infection within 12 days (Fig. 4A). Increasing the challenge dose to 2 × 10^5^ CFU resulted in 100% of mice succumbing within 3 days (Fig. 4B). K96243 Δ*dsbB* was significantly attenuated within the mouse model at both challenge doses tested, with 100% survival at the challenge dose of 1.84 × 10^4^ CFU (Fig. 4A) and 60% survival at the higher challenge dose of 1.84 × 10^5^ CFU (Fig. 4B). MSHR2511 Δ*dsbB* was also significantly attenuated compared to the WT strain in the mouse model, with 100% survival of mice at either challenge dose (Fig. 4C and D). For mice challenged with 2.88 × 10^4^ CFU MSHR2511, 60% succumbed within 4 days, while at a challenge dose of 2.88 × 10^5^ CFU, 100% of mice succumbed within 1.5 days.

### A peptide derived from the second periplasmic loop of BpsDsbB binds to the catalytic surface of BpsDsbA.

In order to understand how BpsDsbB structurally engages with BpsDsbA, we cocrystallized BpsDsbA in a covalent complex with a BpsDsbB peptide. BpsDsbA in which the buried active-site cysteine (Cys 46) is mutated to alanine (BpsDsbA_C46A_) was incubated with a synthesized peptide of sequence Gly-Phe-Ser-Cys-Gly-Phe derived from residues 99 to 104 in the predicted periplasmic loop 2 of BpsDsbB. As a result of the active-site mutation, BpsDsbA cannot resolve the resulting intermolecular disulfide bond, creating a long-lived homogeneous complex suitable for crystallization (24). Accordingly, we determined the crystal structure of BpsDsbA in complex with a 6-mer peptide of BpsDsbB (GFSCGF_Bpspep_) derived from periplasmic loop 2 of BpsDsbB (BpsDsbA-GFSCGF_Bpspep_) in space group P1 using molecular replacement methods to 2.5 Å resolution. There are 4 molecules in the asymmetric unit. The model is refined with an *R*_work_ value of 19.7% (*R*_free_ 25.5%), indicating that it represents the data very well. Details of data collection and quality and additional indicators of the quality of the final model are given in Table 1.

**TABLE 1:** Data for X-ray crystal structure of BpsDsbA-GFSCGF_Bpspep_ complex

Parameter	Value(s) for BpsDsbA-GFSCGF_Bpspep_ complex^*b*^
Data collection and processing statistics	
Wavelength (Å)	0.95370
Resolution range (Å)	58.87–2.59 (2.62–2.49)
Space group	P1
Unit cell dimensions	
*a*, *b*, *c* (Å)	56.8, 59.7, 71.7
α, β, γ (°)	89.5, 67.8, 81.0
*R*_merge_ (%)	0.095 (0.375)
*R*_meas_	0.134 (0.530)
Total no. of observations	111,338 (14,316)
Total no. of unique observations	29,114 (3,835)
Mean *I*/SD *I*^*f*^	10.3 (3.0)
Mean *I* CC(1/2)^*a*^	0.993 (0.856)
Completeness (%)	96.6 (87.7)
Multiplicity	3.8 (3.7)
Refinement and model quality statistics	
Refinement statistics	
*R*_factor_ (%)	19.71 (27.17)
*R*_free_^*c*^ (%)	25.50 (34.82)
No. of:	
Non-H atoms	6,410
Protein atoms	6,126
Ligand atoms	0
Water molecules	284
No. of protein residues	781
RMSD from ideal geometry	
Bond length (Å)	0.002
Bond angle (°)	0.50
MolProbity analysis	
Ramachandran plot (%)	
Favored regions	97.65
Allowed regions	2.35
Outliers	0.00
Rotamer outliers	0.77
Clash score^*d*^	0.82
MolProbity score^*e*^	0.83
B factor (Å^2^)	
Avg	31.4
For protein atoms	31.6
For solvent molecules	27.4

a *I*, intensity; CC(1/2), correlation coefficient between intensity estimates from half data sets.

b Values in parentheses refer to the highest-resolution shell.

c *R*_free_ calculated over 5.0% of the total reflections excluded from refinement.

d The clash score is the number of serious clashes per 1,000 atoms. The value of 0.82 is the 100th percentile value (compared among 269 structures in the resolution range of 2.490 ± 0.25 Å), where the 100th percentile value is the best among structures of comparable resolution and the 0th percentile is the worst. For the clash score, the comparative set of structures was selected in 2004.

e The MolProbity score combines the clash score, rotamer, and Ramachandran evaluations into a single score, normalized to be on the same scale as X-ray resolution. The value of 0.83 is the 100th percentile value (compared among 6,897 structures in the resolution range of 2.49 ± 0.25 Å), where the 100th percentile value is the best among structures of comparable resolution and the 0th percentile is the worst. For the MolProbity score, the comparative set of structures was selected in 2006.

f Mean *I*/SD *I* is the mean intensity divided by the error.

**FIG 5:**
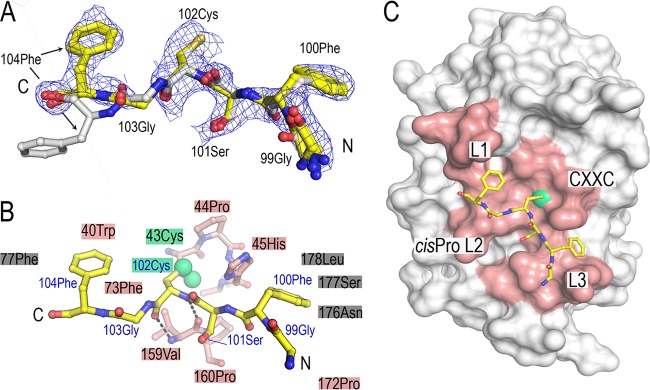
Crystal structure of BpsDsbA in complex with a 6-mer peptide derived from periplasmic loop 2 of BpsDsbB (GFSCGF_Bpspep_). (A) The conformations of the four chains in the asymmetric unit are superimposed. The binding mode is very similar in all four complexes in the asymmetric unit, with the exception of the C-terminal end of chain G (carbons colored white), where the side chain of 104 Phe is oriented away from the surface of BpsDsbA. The 2m*F_o_* − D*F_c_* map is displayed at a 1-sigma contour level for GFSCGF_Bpspep_ (chain F), where 2m*F_o_* − D*F_c_* is a sigma A weighted density map; *F_o_* and *F_c_* are the experimentally measured and model-based amplitudes, respectively; m is the figure of merit; and D is the sigma A weighting factor. (B) The BpsDsbB peptide is shown in stick representation and colored yellow. BpsDsbA residues forming the binding interface with the peptide are indicated with shaded boxes or shown as sticks (specifically, 43 Cys-45 His of the active-site CXXC loop and 159 Val-160 Pro of the *cis*-Pro loop [L2]). BpsDsbA residues which contact the peptide are colored salmon if they make up the active-site surface loops and gray if not. The active-site cysteine 43 is highlighted in green, and the sulfur atoms of both BpsDsbA 43 Cys and peptide 102 Cys are shown as green spheres. GFSCGF_Bpspep_ 102 Cys_Bpspep_ makes two main-chain-mediated hydrogen bonds to *cis*-Pro-1 residue 159 Val of BpsDsbA, indicated by dashed black lines. (C) GFSCGF_Bpspep_ binds on the catalytic surface of BpsDsbA, engaging each of the four loop regions that comprise the active-site face of the protein: the active-site CXXC motif (43 Cys-44 Pro-45 His-46 Cys) and loops linking B3 and H2 (L1), H6 and B4 (L2), and B5 and H7 (L3) (reviewed in references 13 and 25). The BpsDsbA surface showing the region comprising the four loops is colored salmon, with the active-site cysteine (Cys 43) colored green.

The four peptides in the asymmetric unit superimpose upon each other with a high degree of agreement (root mean square deviation [RMSD], 0.15 to 0.59 Å between 6 equivalent C-α atoms), and each adopts a similar conformation on the surface of its respective BpsDsbA molecule. There is a single significant deviation of the C-terminal residues of one protomer (chain G), where the main chain is oriented differently such that the side chain of Phe 104 (which lacks electron density) would project away from the surface of the protein rather than being buried in a hydrophobic pocket, as it is in each of the three other protomers (Fig. 5A). Here, description of the structure is restricted to protomer 1 (chain A [BpsDsbA] and chain F [peptide]).

The active-site surface of DsbA enzymes comprises four loop regions that govern enzymatic activity, redox character, and interactions with substrate and partner proteins. These are the active-site CXXC motif (43 Cys-44 Pro-45 His-46 Cys in BpsDsbA) and loops linking B3 and H2 (loop 1 [L1]), H6 and B4 (loop 2 [L2]), and B5 and H7 (loop 3 [L3]) (reviewed in references 13 and 25). L2 contains a highly conserved *cis*-Pro residue which is positioned adjacent to the CXXC motif. L3 participates in partner protein binding (26). GFSCGF_Bpspep_ makes contacts with each of BpsDsbA's four catalytic surface loops (Fig. 5B and C). Its interaction with BpsDsbA is anchored by a central disulfide bond to the active site, and the flanking hydrophobic pocket contacts both Bpspep phenylalanine residues. Specifically, 102 Cys of the B. pseudomallei GFSCGF peptide (102 Cys_Bpspep_) is disulfide bonded to 43 Cys (2.04 Å) of BpsDsbA (Fig. 5B). 102 Cys_Bpspep_ also makes two main-chain-mediated hydrogen bonds with the *cis*-Pro-1 residue 159 Val of L2 (Fig. 5B). 100 Phe_Bpspep_ is sandwiched in a pocket between BpsDsbA residues 176 Asn, 177 Ser, and 178 Leu (L3) and the side chain of the active-site residue BpsDsbA 45 His. 178 Leu forms the wall of this pocket, providing a favorable hydrophobic character to accommodate the phenyl ring of 100 Phe_Bpspep_. Similarly, the phenyl ring of 104 Phe_Bpspep_ inserts into a small hydrophobic pocket lined with BpsDsbA 73 Phe, 77 Phe, and 40 Trp. This effectively flanks the central disulfide bond with N- and C-terminal hydrophobic contacts. 99 Gly_Bpspep_'s and 103 Gly_Bpspep_'s carbonyl oxygens and 101 Ser_Bpspep_'s side chain hydroxyl are surface exposed and make no specific hydrogen bond contacts with the surface of BpsDsbA (Fig. 5B and C). In three protomers of the asymmetric unit, 47 to 65% of the DsbA residues forming the interface (identified using the PISA tool [27]) make hydrophobic contacts with the peptide. For the fourth protomer involving the differently oriented chain G (Fig. 5A), this falls to 40%.

As observed for the apo and the E. coli DsbB-derived heptamer peptide PFATCDS (PFATCDS_Ecpep_)-bound structures of EcDsbA (24), BpsDsbA accommodates peptide binding with few and minor conformational changes. BpsDsbA-GFSCGF_Bpspep_ superimposes on apo WT BpsDsbA with an RMSD of 0.3 Å over 189 equivalent C-α atoms, and the catalytic surface loops are equivalently positioned. Critically, the active-site backbone is essentially identical between the WT and the BpsDsbA_C46A_ variant, indicating that this mutation does not induce local conformational changes. In BpsDsbA-GFSCGF_Bpspep_, the side chain of active-site residue 45 His is rotated relative to its position in the apo WT BpsDsbA, thus accommodating 100 Phe_Bpspep_ in the complex structure. Equivalent active-site His residues have been observed to be mobile in other DsbA structures (e.g., Pseudomonas aeruginosa DsbA [28]).

The surface area of the BpsDsbA-GFSCGF_Bpspep_ interface is 445 Å^2^ with a shape complementarity score of 1.0 (calculated using the PISA tool [27]). This compares to an interface area of 784.3 Å^2^ and a shape complementarity score of 0.836 for EcDsbA and EcDsbB and is consistent with the hypothesis that DsbA-DsbB protein-protein interactions may be typified by relatively small surface areas but high shape complementarity (26).

### GFSCGF_Bpspep_ binds to BpsDsbA in a manner similar to that in which PFATCDS_Ecpep_ binds to EcDsbA.

**FIG 6:**
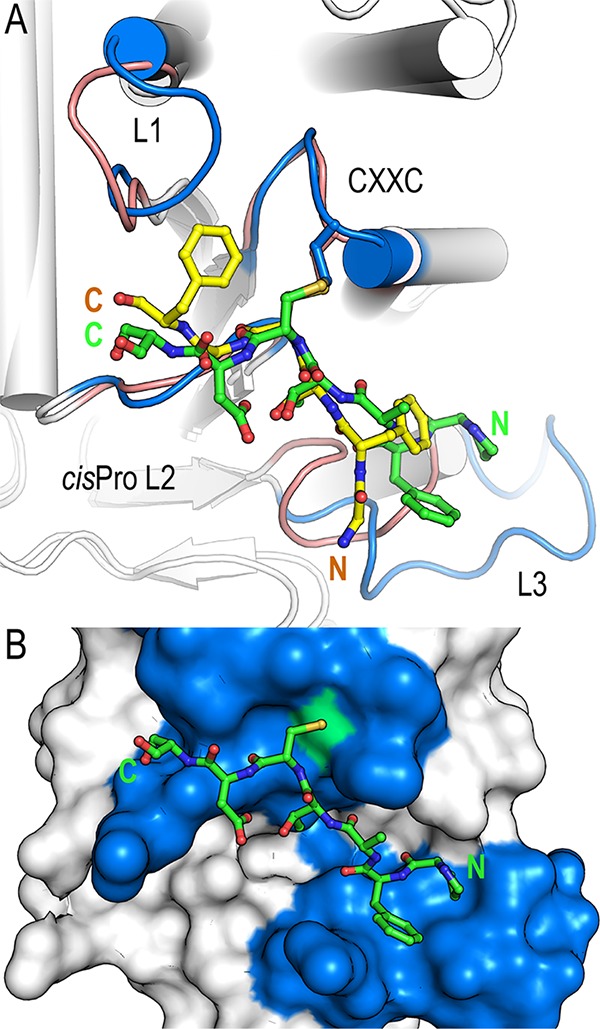
Comparison of BpsDsbA in complex with GFSCGF_Bpspep_ and E. coli DsbA in complex with the E. coli DsbB-derived heptamer peptide PFATCDS_Ecpep_. (A) Comparison of EcDsbA-PFATCDS (PDB accession number 4TKY [24]) and BpsDsbA-GFSCGF. The crystal structure of each complex is shown in cartoon representation, with helices represented by cylinders. Active-site loops are labeled and highlighted for EcDsbA (blue) and BpsDsbA (salmon), and the respective bound peptides are shown as sticks (PFATCDS_Ecpep_ in green and GFSCGF_Bpspep_ in yellow). The C-terminal sections of GFSCGF_Bpspep_ and PFATCDS_Ecpep_ superimpose closely, and the cysteine of each peptide and adjacent main-chain atoms superimpose almost exactly. In contrast, the two peptides deviate significantly at their N termini, with a distance of up to 5 Å between the C-α of the equivalent residues 99 Gly_Bpspep_ and 101 Phe_Ecpep_. This deviation is a consequence of the shorter loop L3 in BpsDsbA than in EcDsbA. (B) In EcDsbA, a longer L3 loop forms an extended groove immediately beneath the active site to accommodate the N-terminal proline and phenylalanine. EcDsbA is shown in surface representation. Coloring is as described in the legend to panel A. The active-site cysteine (EcDsbA Cys 33) is highlighted in green. The N and C termini of the peptides are labeled in each panel.

BpsDsbA is classified as a DsbA-Ib enzyme, while EcDsbA is a DsbA-Ia enzyme with a catalytic surface characterized by a significant hydrophobic binding groove beneath the active site (25). In crystal structures of EcDsbA-EcDsbB and EcDsbA with a EcDsbB periplasmic loop peptide mimetic, EcDsbB or peptide has bound within this groove (24, 26). We hypothesized that as BpsDsbA has a very different active-site landscape featuring small discrete pockets rather than an expansive groove, engagement with a peptide or with BpsDsbB may generate a binding mode distinct from that seen in EcDsbA complexes (19, 25). To address this question, we compared the structures of EcDsbA-PFATCDS_Ecpep_ (PDB accession number 4TKY) and BpsDsbA-GFSCGF_Bpspep_. We found that GFSCGF_Bpspep_ and PFATCDS_Ecpep_ bound in a very similar manner across the catalytic surface of BpsDsbA and EcDsbA, respectively, with each engaging all four active-site surface loops (Fig. 6A).

Superposition of the two complexes shows that the C-terminal sections of GFSCGF_Bpspep_ and PFATCDS_Ecpep_ superimpose closely. As expected, and reflecting the commonality of disulfide bonding, the cysteine of each peptide and adjacent main-chain atoms superimpose almost exactly. In contrast, the two peptides deviate quite significantly at their N termini, with a distance of up to 5 Å between the C-α of the equivalent residues 99 Gly_Bpspep_ and 101 Phe in the E. coli peptide (Fig. 6A). This marked deviation is a consequence of the contracted length of L3 in BpsDsbA relative to EcDsbA; in EcDsbA, a longer L3 (linking B5 and H7) creates a sizeable pocket beneath the active site to accommodate 100 Pro-101 Phe (Fig. 6A and B); in BpsDsbA, a significantly shorter L3 turn does not (Fig. 6A and 5C).

## DISCUSSION

Burkholderia pseudomallei is naturally resistant to many commonly used antibiotics, including penicillins, first- and second-generation cephalosporins, aminoglycosides, and polymyxins (2). International guidelines for treatment advise a minimum 10 to 14 days of intravenous antibiotics (intensive period) followed by 3 to 6 months of oral antibiotics (eradication period) (2, 12, 29). Adherence during the long eradication phase is important to prevent relapse (30), and in Australia, a substantial proportion of patients fail to complete the second eradication therapy (only 50% of 215 patients) (29). If the intensive period is extended to ∼25 days, relapse is rare even if patients fail to adhere to the eradication therapy (29, 31). Longer intensive periods are not always feasible. For example, in Laos even 10 days of ceftazidime administration is problematic because of both cost and patient circumstances (32). Shorter courses of more effective antimicrobials that lower mortality, shorten hospitalizations, and lessen outpatient adherence requirements would improve patient outcomes on a global scale.

Bacterial DSB proteins are emerging as a promising new avenue for antivirulence therapeutics (33). We have demonstrated here that B. pseudomallei DsbB is the partner protein of the known virulence regulator BpsDsbA (19) and contributes to virulence in a mouse model of melioidosis. Deletion of *dsbB* from B. pseudomallei K96243 and MSHR2511 protected mice from lethal peritoneal infection, demonstrating that BpsDsbB is a potential drug target for antimicrobial development. Furthermore, the protein sequence of BpsDsbB is highly conserved among 431 diverse clinical isolates of B. pseudomallei, underpinning the importance of this enzyme in the bacterium and suggesting that BpsDsbB inhibitors could be broadly effective in melioidosis. The observed mutations in BpsDsbA do not map to the active-site surface loops of the protein structure (Fig. 1) and thus are not predicted to alter protein function. The structure of BpsDsbB has not yet been determined. Three of the four observed mutations are predicted to be located on a transmembrane helix or to face the cytoplasm (Fig. 1). A fourth mutation (Arg118His) is predicted to be in the second periplasmic loop. This mutation has the potential to alter interactions with BpsDsbA, but we note that the modification is conservative and maintains a positively charged side chain.

*dsbB* deletion likely impacts B. pseudomallei virulence because it interrupts the normal redox activity of BpsDsbA. DsbA oxidatively folds disulfide bonds in substrate proteins and is itself reduced in the process. DsbB in turn accepts electrons from DsbA to restore it to its oxidized primed state, ready for the next substrate folding event. Consistent with this, the phenotype of K96243 Δ*dsbB* mimics the *in vivo* phenotype of K96243 Δ*dsbA* in our infection model and broadly mimics the *in vitro* K96243 Δ*dsbA* phenotypes assayed, although we note that the magnitude of the effect on protease secretion and motility is reduced relative to that observed in K96243 Δ*dsbA*. This recapitulation of the Δ*dsbA* mutant phenotypes is consistent with a BpsDsbB-mediated loss of BpsDsbA activity in the current study.

We also showed that both the K96423 Δ*dsbB* and MSHR2511 Δ*dsbB* strains are highly attenuated in an animal model of infection. Surprisingly, these *dsbB* deletion strains exhibited very different effects in two standard *in vitro* virulence assays. This suggests that the *in vitro* experiments, which examine just two hallmarks of virulence, do not fully reflect the impact of BpsDsbB on virulence. We determined that deletion of *dsbB* does not affect the growth of B. pseudomallei *in vitro* under either aerobic or anaerobic conditions; wild-type and mutant growth curve profiles were equivalent, and there was no intrinsic growth defect caused by the gene deletion (see Fig. S1 in the supplemental material). Disruption of the DsbA/DsbB pathway is likely to impact many disulfide bond-containing substrates, and the assessment of just two phenotypes does not characterize the broader impact of *dsbB* disruption. This highlights the challenge of identifying the mechanism(s) by which *dsbB* deletion leads to a reduction in virulence, as changes to virulence are likely to be the consequence of disruption of multiple pathways, potentially across time. A future study to unpick the precise impact of *dsbB* deletion on B. pseudomallei infectivity, elicitation of an inflammatory response, and bacterial dissemination would be of great interest in light of reports that deletion of individual DSB proteins in a number of species results in pleiotropic phenotypes *in vitro*, including alterations to extracellular enzyme formation (34), adhesion (35), toxin production and secretion (36), competence (37), and antibiotic sensitivity (22). *In vivo*, the additional environmental and immune system stresses encountered by the bacteria within the host add further complexity to understanding how *dsbB* loss disrupts virulence. For example, quantification of bacterial numbers in infected organs or macrophages can report changes in bacterial load but does not distinguish between a growth defect (though our *in vitro* growth curve experiments do not suggest this would be the case for Δ*dsbB* bacteria) and a reduced capacity to survive and disseminate in the host environment. The loss of pathogenicity in the animal model reported here is conclusive evidence of the key role played by BpsDsbB in virulence *in vivo*, although the precise pathway(s) by which the observed attenuation is achieved is yet to be elucidated. In future, it will also be important to analyze a panel of strains, as we have done, and to investigate the effect of deletions in animal models of infection to establish roles for virulence and to test potential therapeutics. The phenotypic range observed in the *in vitro* assays likely also reflects the known diversity of the B. pseudomallei species (38, 39, 40).

There has recently been significant progress in the identification of inhibitors of DsbB and DsbB-like bacterial proteins (33). The DsbB-like protein vitamin K epoxide (VKOR) in Mycobacterium tuberculosis can be inhibited by warfarin (41). Inhibitors of E. coli DsbB have been identified by fragment-based screening using purified protein (50% inhibitory concentration [IC_50_], 7 to 200 μM) (42). These were later advanced to an IC_50_ of ∼1 μM with a rational medicinal chemistry program (43). Additional EcDsbB inhibitors resulted from high-throughput screening in E. coli cells (44). Significantly, the lead compound in this study also inhibits DsbB proteins from other bacterial species. Together these data indicate that DsbB can be effectively inhibited and that successful compounds can have broad-spectrum activity.

The 2.5-Å-resolution structure of a BpsDsbB peptide in complex with BpsDsbA that we report provides an important framework for future rational efforts to identify and develop small-molecule inhibitors of BpsDsbA. This is the fourth reported structure of a DsbA in a covalent (disulfide or thioether) complex with a peptide, joining EcDsbA in complex with a DsbB-derived peptide (PDB accession number 4TKY [24]), EcDsbA in complex with a substrate peptide from SigA (PDB accession number 3DKS [45]), and Xylella fastidiosa DsbA in complex with a peptide of unknown sequence (PDB accession number 2REM [46]). The peptide cysteine (or homoserine; PDB accession number 3DKS) and the flanking residues Cys −1 and Cys +1 are very similarly positioned in each structure. The peptide cysteine is engaged in a disulfide bond with the N-terminal active-site Cys. It also makes two conserved main-chain hydrogen bonds to main-chain atoms of the neighboring *cis*-Pro-1 residue. Note that in the EcDsbA-SigA complex the disulfide bond is replaced by a thioether bond and one of the main-chain bonds to *cis*-Pro-1 is water mediated, but the mode of interaction is highly similar to that observed in the disulfide-bonded DsbA-peptide complexes. There are a very limited number of DsbA-peptide structures from which to draw broad conclusions, but nevertheless, it seems that the conserved required DsbA-binding motif is minimal: a disulfide bond, and two hydrogen bonds between the backbone atoms of the incoming Cys and the DsbA *cis*-Pro-1 residue. Small molecules or peptidomimetics that block this site may inhibit BpsDsbA-BpsDsbB interaction and activity, though specificity with respect to other thioredoxin fold proteins would also need to be considered. On this point, we note that small-molecule inhibitors of EcDsbA have been reported (47). These inhibitors recapitulate the hydrophobic interactions observed between an EcDsbB-derived peptide and the EcDsbA active-site surface groove, have inhibitory activity in a model oxidative folding assay, and disrupt E. coli cell motility, presumably by disturbing productive interactions between EcDsbA and EcDsbB and/or substrates.

DSB enzymes are not essential for aerobic growth in nutrient-rich media, and DsbA- and DsbB-deficient B. pseudomallei strains show no loss of viability or growth rate under these conditions (19; the present study). Antimicrobials that inhibit DSB proteins under aerobic conditions would thus be antivirulence agents rather than antibiotics. The potential advantages of antivirulence agents are manifold (48). Antivirulence agents do not kill bacteria or prevent their growth, so it is likely that they will exert a reduced selection pressure for development of resistance (49), and as they target virulence effectors, they may spare commensal bacteria. New antibiotic discovery is slow, and just five of the antibiotics approved since 2000 have been first in class (50). New antimicrobials with a novel mode of action are urgently required to reinvigorate our antimicrobial resources. Antivirulence drugs, used in combination with standard antimicrobials, may revitalize current therapeutics no longer deemed clinically useful due to widespread resistance.

## MATERIALS AND METHODS

### Bacterial strains and growth conditions.

Five B. pseudomallei strains were selected for the current work on the basis of their diverse genetic and clinical backgrounds: two clinical Thai isolates, K96243 (51) and 576 (52), and three Australian clinical isolates, MSHR0305b (53), MSHR2511, and MSHR5858 (54, 55). MSHR0305b is from patient P101, a nondiabetic male with fatal neurological melioidosis. Importantly, P101 had a mixed infection with two genetically disparate strains, MSHR305 (56) and MSHR0305b. MSHR2511 was from a nondiabetic male with rapidly fatal pulmonary melioidosis. MSHR5858 is from a male diabetic patient (patient P730) who presented with acute pulmonary melioidosis. P730 ultimately survived his infection. MSHR5858 was chosen for this study because it belongs to sequence type 562, a group of strains isolated in Australia but hypothesized to be a recent introduction into Australia from Asia (57). Strains 576 and MSHR0305b contain lipopolysaccharide (LPS) type B, whereas strains MSHR2511, MSHR5858, and K96243 contain LPS type A. B. pseudomallei isolates were grown on Ashdown's agar (Oxoid), chocolate agar (Oxoid), or LB agar at 37°C under aerobic conditions.

### Ethics statement.

Bacterial samples were obtained from the Darwin Prospective Melioidosis Study bacterial isolate collection, with ethical approval obtained through the Human Research Ethics Committee of the Northern Territory Department of Health and the Menzies School of Health Research (approval no. HREC 02/38, Clinical and epidemiological features of melioidosis). All patient data, with the exception of clinical presentation/severity, were deidentified prior to analysis.

### Construction of *dsbB* knockouts.

The *dsbB* gene was removed from the five B. pseudomallei clinical isolates using the pMo series of vectors and the methodology described previously (58). The plasmids and strains and the primers used in the current study are detailed in Tables 2 and 3, respectively. Real-time PCR targeting a conserved region of *dsbB* was used to verify the knockouts. Three (K96243, MSHR0305b, and MSHR2511) of the five isolates were subjected to whole-genome sequencing to verify the complete removal of *dsbB* and that no additional mutations had been introduced during the knockout process.

**TABLE 2:** Bacterial strains and plasmids used in this study

Strain or plasmid	Description	Reference or source
Strains		
B. pseudomallei MSHR0305b	Australian clinical isolate from patient P101	This study; 56
B. pseudomallei MSHR2511	Australian clinical isolate from patient P498	This study
B. pseudomallei MSHR5858	Australian clinical isolate from patient P730	54
B. pseudomallei K96243	Thai clinical isolate	51
B. pseudomallei 576	Thai clinical isolate	52
B. pseudomallei MSHR0305b Δ*dsbB*	Δ*dsbB* knockout	This study
B. pseudomallei MSHR2511 Δ*dsbB*	Δ*dsbB* knockout	This study
B. pseudomallei MSHR5858 Δ*dsbB*	Δ*dsbB* knockout	This study
B. pseudomallei K96243 Δ*dsbB*	Δ*dsbB* knockout	This study
B. pseudomallei 576 Δ*dsbB*	Δ*dsbB* knockout	This study
Escherichia coli DH5α	General cloning strain	78
E. coli S17-1	Plasmid mobilization strain	79
Plasmids		
pMo130	Suicide vector for allelic exchange in Burkholderia spp.; *xylE sacB* Kan^r^	58
pMo130-dsbB-US-DS	Suicide vector used to construct *dsbB* knockouts	This study

**TABLE 3:** Primers used for construction of *dsbB* knockout strains and for verification of *dsbB* removal

Primer purpose and primer	Sequence (5′–3′)^*a*^
Allelic exchange	
DsbB_US_Nhe_F	CGCCGCGCTAGCCGGACTGACGGGCTTCAAG
DsbB_US_Bgl_R	TTAACGAGATCTTCGCGCCGCTTTTC
DsbB_DS_Bgl_F	TGATCGAGATCTAATAGCCCGCATCATTG
DsbB_DS_HindIII_R	TCGCCTAAGCTTCGAGATGACCGCCGCCG
Knockout confirmation	
DsbB_PA_F	TCTGCAGTACGTGAAAAACGAGGA
DsbB_PA_R	CCGGGGTTCAACTGCACGTA

a Underlined sequences indicate restriction enzyme sites for downstream cloning.

We also made several efforts to complement the Δ*dsbB* deletion mutants but were unsuccessful. First, we introduced the *dsbB* gene on a multicopy plasmid into the deletion mutants, but this did not restore the protease deficiency of the mutant strains. Possible explanations for this inability to complement include the fact that the gene is not expressed to adequate levels from the plasmid, although this is unlikely in a multicopy plasmid. Conversely, the potential overexpression of *dsbB* may have negatively impacted the *dsb* system or the processing of DsbB. We therefore also attempted to complement a single gene copy into the chromosome, but this, too, was unsuccessful. Efforts to chemically complement the wild-type phenotype of the Δ*dsbB* deletion mutants using cystine (22, 23) as a small-molecule oxidizing agent in defined medium rescued a protease secretion phenotype for the K96243 Δ*dsbB* strain, although the effect was modest (data not shown). Whole-genome sequencing of the three Δ*dsbB* isolates (K96243, MSHR0305b, and MSHR2511) verified that no other mutations had occurred in the strains outside of the *dsbB* region. Inspection of neighboring regions excluded the likelihood of polar effects of *dsbB* removal. We note that efforts to complement a B. pseudomallei Δ*dsbA* strain were also unsuccessful (19).

### Whole-genome sequencing and comparative genomic analysis.

Genomic data were generated from paired-end Illumina reads using the HiSeq 2000 platform at the Australian Genome Research Facility (AGRF; Melbourne, Australia) or Macrogen, Inc. (Geumcheon-gu, Seoul, Republic of Korea). Analysis was performed with the SPANDx (version 3.0) pipeline (59), which wraps the BWA (60), SAMtools (61), GATK (62), and SnpEff (63) tools to identify genetic variants in comparison to a reference genome. Genomes were assembled using the Microbial Genome Assembly pipeline (https://github.com/dsarov/MGAP-Microbial-Genome-Assembler-Pipeline).

### BpsDsbB expression, membrane preparation, and purification.

*B. pseudomallei dsbB* (UniProt accession number Q63RY4) and a variant in which four cysteine residues predicted to be in the periplasmic loops were mutated to serine (Cys41Ser, Cys44Ser, Cys102Ser, and Cys130Ser [BpsDsbB_SSSS_]) were each inserted into a pET28a vector with a noncleavable C-terminal His_8_ tag. BpsDsbB and BpsDsbB_SSSS_ were expressed in E. coli C41 cells in PASM autoinduction medium (64) with kanamycin (50 μg/ml) (16 h, 30°C, 220 rpm). Harvested cells were resuspended in phosphate-buffered saline (PBS; pH 7.4; 200 ml/liter culture) with DNase and protease inhibitors and lysed using a constant-pressure cell disrupter (two successive passages at 27,000 and 30,000 lb/in^2^, respectively). Large cellular debris was removed by centrifugation (15,000 × *g*, 4°C, 15 min). Membranes were isolated from the resulting supernatant by ultracentrifugation (185,000 × *g*, 1 h 15 min, 4°C) and homogenized in 40 ml of PBS. For purification, membranes were solubilized in PBS and 1% (wt/vol) *n*-dodecyl-β-d-maltopyranoside (DDM; Glycon Biochemicals) with vigorous stirring (1 h, 4°C). Detergent-solubilized protein was isolated by ultracentrifugation (170,000 × *g*, 1 h 15 min, 4°C), adjusted to a final concentration of 40 mM imidazole, and applied to a 5-ml HisTrap nickel affinity column (GE Healthcare) equilibrated in PBS, 40 mM imidazole, 10% (vol/vol) glycerol, and 0.3% *n*-decyl-β-d-maltopyranoside (DM; Glycon Biochemicals). The column was washed with 10% glycerol, 0.3% (wt/vol) DM in PBS (buffer 1) plus 40 mM imidazole (20 bed volumes [bv] buffer 1 plus 60 mM imidazole [20 bv]). BpsDsbB was eluted in buffer 1 plus 0.5 M imidazole, followed by a final polishing size exclusion chromatography step (Superdex 200 16/60) in 25 mM MES (morpholineethanesulfonic acid; pH 6.5), 150 mM NaCl, 0.15% (wt/vol) DM. Protein purity was assessed by SDS-PAGE.

### BpsDsbA expression and purification.

Codon-optimized *B. pseudomallei dsbA* (UniProt accession number Q63Y08) and a variant in which Cys 46 was mutated to alanine (C46A) were each inserted into a modified pET21a vector with a tobacco etch virus (TEV) protease-cleavable N-terminal His_6_ tag as described in reference 19. BpsDsbA and BpsDsbA_C46A_ were expressed and purified as described previously (19) with modifications. Harvested cells were resuspended in 25 mM HEPES, pH 7.5, 150 mM NaCl (buffer 2), DNase, and protease inhibitors and lysed using a constant-pressure cell disrupter (18,000 ln/in^2^). Clarified lysate was adjusted to a final concentration of 5 mM imidazole and purified with Talon resin (Clontech, Australia) equilibrated in buffer 2 with 5 mM imidazole, washed with 25 mM HEPES, pH 7.5, 500 mM NaCl, 10 mM imidazole (5 bv), buffer 2 plus 10 mM imidazole (5 bv), and eluted with buffer 2 plus 500 mM imidazole (5 bv). Purified protein was exchanged into buffer 2 using a column packed in-house with Sephadex G-25 resin, to remove imidazole prior to cleavage and removal of the N-terminal His_6_ tag (described in reference 18) prior to a final size exclusion step in buffer 2 (Superdex S75 16/60 column).

### Oxidation and reduction of DSB proteins.

B. pseudomallei and E. coli DsbA proteins were reduced with 10 mM dithiothreitol (DTT; reduced) or oxidized with 20 mM oxidized glutathione (GSSG). Samples were incubated for at least 30 min on ice. Redox agents were removed from the protein preparations using a column (a PD-10 desalting column or an S75 16/60 size exclusion chromatography column) equilibrated in degassed buffer without redox agents. An Ellman assay was performed to verify the protein redox state (65).

### BpsDsbA-mediated oxidative folding assay.

B. pseudomallei DSB redox activity was assessed using an oxidative folding assay as described previously (19, 43) in which DSB-catalyzed folding of a model peptide is monitored fluorometrically over time. BpsDsbA (80 nM) and 1.6 μM either WT BpsDsbB or BpDsbB_SSSS_ (crude membrane extracts) were combined with peptide substrate at a final concentration of 8 μM. Plotted data show the means and standard deviations (SDs) for four (WT BpsDsbB) or two (BpsDsbB_SSSS_) biological replicates.

### BpsDsbB-catalyzed reduction of ubiquinone.

We assessed the ability of purified detergent-solubilized DsbB to reduce ubiquinone-1 (UQ1; coenzyme Q1; Sigma) in the presence of DsbA using a Cary 50 UV-visible spectrophotometer (Varian) (14, 66). Reduction of UQ1 is accompanied by a decrease in its absorbance at 275 nm. The assay was performed in a final volume of 100 μl at 30°C in a reaction mixture of 30 μM reduced BpsDsbA (BpsDsbA_reduced_), 1 μM BpsDsbB, and 45 μM UQ1 in 25 mM MES, 150 mM NaCl, 0.3% (wt/vol) DM, pH 6.5. Control reactions omitted BpsDsbA, BpsDsbB, or UQ1. The reaction mixtures with E. coli proteins had 30 μM EcDsbA_reduced_, 80 nM EcDsbB, and 45 μM UQ1. Three independent experiments were conducted, and representative data from one of these independent experiments are reported.

### Redox reactions between BpsDsbA and BpsDsbB.

The ability of purified detergent-solubilized DsbB to oxidize reduced DsbA was assessed by alkylation with AMS (4′-acetamido-4′-maleimidylstilbene-2,2′-disulfinic acid) and protein separation by SDS-PAGE. A 200-μl reaction mixture of 30 μM BpsDsbA_reduced_, 0.5 μM BpsDsbB, and 45 μM UQ1 in 25 mM MES, 150 mM NaCl, 0.3% DM, pH 6.5, was incubated with gentle mixing at 30°C. After 5 and 30 s and 1, 2, 5, 10, and 30 min, 20-μl samples were mixed with 40 μl of 10% (wt/vol) trichloroacetic acid (TCA) to stop the reaction and precipitate the proteins. Samples were incubated on ice for 15 min and then centrifuged (18,000 × *g*, 10 min), and the supernatant was decanted. The pellet was washed (200 μl of 100% ice-cold acetone) and centrifuged again (18,000 × *g*, 10 min), the acetone was decanted, and the tubes were air dried for 30 min. The pellets were mixed with 20 μl of 5 mM AMS in 1% SDS, 50 mM Tris, pH 7.0, and 20 μl of loading dye (without reducing agent). Five microliters of this mixture was analyzed on a 12% bis-Tris gel (NuPAGE; Invitrogen) (100 V in MOPS [morpholinepropanesulfonic acid] buffer [Novex, Life Technologies], 180 min). For E. coli protein reference reactions, 2.5 μl of the final sample was analyzed by SDS-PAGE. Experiments were repeated on three independent occasions, and representative data from one of those experiments are reported.

### *In vitro* growth analysis of B. pseudomallei Δ*dsbB* strains.

B. pseudomallei strains were initially grown overnight in 50 ml LB broth in 250-ml flasks at 180 rpm and 37°C. Aerobic growth curves were initiated by adjusting the OD_590_ of 50 ml of LB to 0.10 with the appropriate dilution of overnight culture. The OD_590_ was recorded hourly, and the experiment was repeated on three separate occasions. Anaerobic cultures were adjusted to an OD_590_ of 0.10 in 50 ml of LB containing 0.75% glucose and 25 mM sodium nitrate (21). Each flask was incubated at 37°C and 180 rpm in O-ring-sealed biojars containing GENbag Anaer gas packs (bioMérieux, Basingstoke, United Kingdom). A separate flask was prepared for each time point, and the experiment was repeated on two separate occasions. Statistical analysis was performed by two-way analysis of variance (ANOVA) following log transformation of the data.

### Protease assay.

An azocasein hydrolysis assay was performed as previously described with modification (19, 67). B. pseudomallei strains were grown from glycerol stocks in LB broth for 18 h at 37°C and 200 rpm. Cells were harvested by centrifugation and resuspended in M9 minimal medium, the bacterial concentration was adjusted to 5 × 10^7^ CFU/ml by measurement of the optical density, and the culture was incubated for 18 h at 37°C and 200 rpm. The supernatant from each culture was prepared by centrifugation for 15 min at 13,000 rpm, and the pellet was discarded. The protease assay was performed by adding 125 μl of supernatant to 125 μl of azocasein (5 mg/ml) prepared in 0.05 M Tris-HCl, pH 8.0. The reaction mixture was incubated at 37°C for 2.5 h and stopped by the addition of 250 μl 10% TCA, followed by centrifugation for 15 min to remove insoluble azocasein. The absorbance at 440 nm was measured following the addition of 500 μl of 0.5 M NaOH. Mean values from three biological replicates with standard errors of the means were calculated. A two-way ANOVA was followed by Sidak's multiple-comparison test to assess significance.

### Motility assay.

B. pseudomallei strains were grown from glycerol stocks and adjusted to 5 × 10^7^ CFU/ml as described above for the protease assay. Each strain (5 μl) was stab inoculated into the center of a semisolid motility agar plate (0.3% LB agar). Motility assay plates were incubated at 37°C for 20 h, and the zones of motility were measured. Mean values from three biological replicates with standard errors of the means were calculated. A two-way ANOVA was followed by Sidak's multiple-comparison test to assess significance.

### Animals.

Age-matched female BALB/c mice, approximately 6 weeks old, were obtained from Charles River (Margate, United Kingdom). The mice had access to food and water and were grouped together in cages of five mice each with 12-h light and 12-h dark cycles. The animals were handled under biosafety level III containment conditions within a half-suit isolator, compliant with British Standard BS5726. All procedures within this study were carried out according to the requirements of the Animal (Scientific Procedures) Act 1986. The bacterial challenge was prepared by suspending LB agar plate-grown B. pseudomallei strains (37°C, 20 h) into LB broth to a concentration of 1 × 10^8^ CFU/ml. The cells were diluted 1/100 and incubated for 18 h at 37°C and 180 rpm. The challenge doses were prepared by dilution to the desired concentration, and 100 μl was used to inoculate animals via the intraperitoneal (i.p.) route of infection. Humane endpoints were strictly observed, and animals assessed to be incapable of survival were humanely killed by cervical dislocation. The animals were monitored twice daily for 42 days. Data were evaluated for statistically significant differences by the log rank (Mantel-Cox) test.

### BpsDsbA-peptide crystal structure determination and refinement.

A peptide (GFSCGF_Bpspep_) was designed on the basis of the sequence flanking the first cysteine (Cys 102) of periplasmic loop 2 in BpsDsbB and synthesized (≥95% purity) by GenicBio Ltd. Lyophilized GFSCGF_Bpspep_ was solubilized in a 0.1 M ammonium bicarbonate, pH 8.0, solution at a final concentration of 15 mM and air oxidized overnight at room temperature with stirring. GFSCGF_Bpspep_ was mixed in an ∼3.4-fold molar ratio with BpsDsbA_C46A_ (final concentrations, 7.5 mM peptide and 2.2 mM protein), and the mixture was incubated (2 h, 20°C). This mixture was buffer exchanged to 25 mM HEPES, pH 7.5, 150 mM NaCl using an Amicon Ultra 0.5-ml centrifugal filter (10-kDa-molecular-mass cutoff), the volume was reduced, and the volume was topped up with the target buffer three times, before final concentration to 64 mg/ml for crystallization. BpsDsbA_C46A_-GFSCGF_Bpspep_ was crystallized using The University of Queensland Remote Operated Crystallization and X-Ray Diffraction Facility and the vapor diffusion method, and hanging drops were dispensed with a Mosquito crystallization robot (TTP Labtech). The temperature of all crystallization experiments was maintained at 293 K. Crystals of BpsDsbA_C46A_-GFSCGF_Bpspep_ (200 nl of 64-mg/ml BpsDsbA_C46A_-GFSCGF_Bpspep_ plus 200 nl of reservoir solution) grew in 0.1 M HEPES, pH 7.0, 0.5% (vol/vol) Jeffamine ED-2001 reagent, and 1.74 M sodium malonate, pH 7.0.

Perfluoropolyether (1 μl; Hampton Research Inc.) was added to the drops as a cryoprotectant, and crystals were flash frozen in liquid nitrogen. Diffraction data were measured at 100 K at the Australian Synchrotron MX2 beamline using an ADSC Quantum 315r detector. Data were collected over a 360° rotation at a wavelength of 0.95370 Å. Using the autoPROC software toolbox (68), the data were indexed and integrated with the XDS program package (69) prior to further processing with the Pointless program and scaling with the Aimless program (70). Inspection of the diffraction images and the proportion of reflections included in the final processing indicated diffraction from more than one lattice in the collected data but just one lattice in the selected data. Diffraction from ice rings was not detected. Following data assessment with the programs Pointless, Aimless, and Xtriage (including probabilistic Matthews coefficient analysis), the space group was determined to be P1 with four copies of BpsDsbA-GFSCGF_Bpspep_ in the asymmetric unit. A high-resolution limit of 2.49 Å was applied to the data following evaluation of the half data set correlation coefficient, *R*_meas_, and completeness values in Aimless. Molecular replacement using BpsDsbA (PDB accession number 4K2D) as a template structure yielded a successful solution in PHASER (71) within the Phenix suite (72). The resulting model was subjected to iterative rounds of refinement (Phenix.Refine [73])—including translation/libration/screw (TLS) parameters and addition of hydrogen atoms using a riding model—and model building using the COOT program (74). The quality of the final model was assessed with MolProbity (75) throughout the refinement process.

### Accession number(s).

The structure of BpsDsbA-GFSCGF_Bpspep_ has been deposited in the Protein Data Bank under accession number 5VYO.

## Supplementary Material

Supplemental material
