# Supplementary material for: Virulence of the Melioidosis Pathogen Burkholderia pseudomallei Requires the Oxidoreductase Membrane Protein DsbB
Source: Infect Immun. 2018 Apr 23;86(5):e00938-17. doi: 10.1128/IAI.00938-17 (PMC5913862; doi:10.1128/IAI.00938-17)
Supplement: Supplemental material [file supp_86_5_e00938-17__index.html]

Supplemental material 

# Virulence of the Melioidosis Pathogen Burkholderia pseudomallei Requires the Oxidoreductase Membrane Protein DsbB

## Supplemental material

- Supplemental file 1 -

  Fig. S1. Growth characteristics of *B. pseudomallei* strains grown aerobically or anaerobically.

  PDF, 459K
