# Supplementary material for: Virulence of the Melioidosis Pathogen Burkholderia pseudomallei Requires the Oxidoreductase Membrane Protein DsbB
Source: Infect Immun. 2018 Apr 23;86(5):e00938-17. doi: 10.1128/IAI.00938-17 (PMC5913862; doi:10.1128/IAI.00938-17)
Supplement: Supplemental material [file IAI.00938-17_zii999092380s1.pdf]

(A)

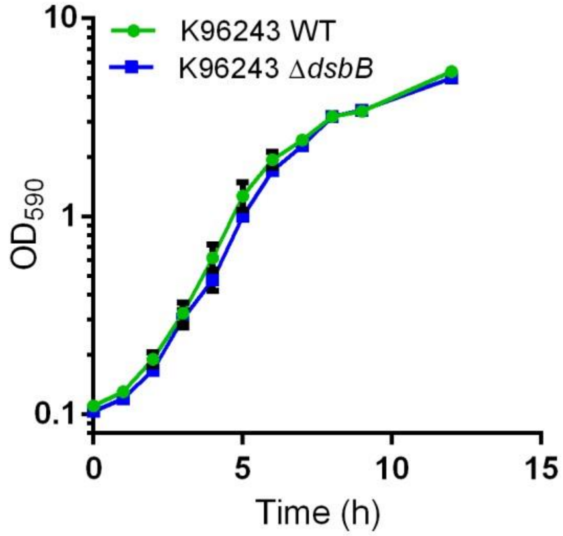

K96243 WT
K96243 ΔdsbB
OD590
10
1
0.1
0
5
10
15
Time (h)

(B)

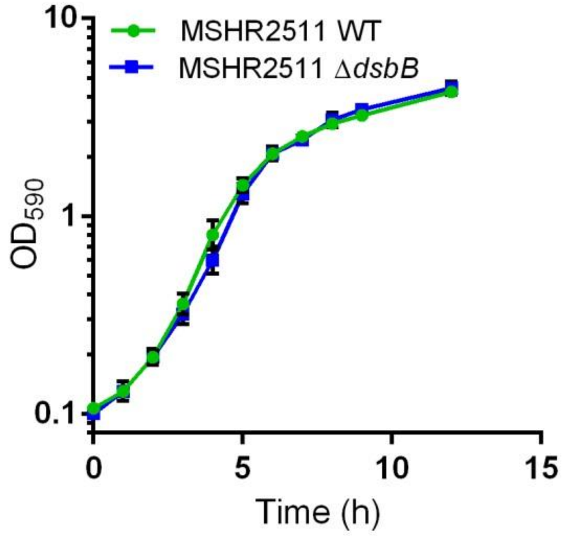

MSHR2511 WT
MSHR2511 ΔdsbB
OD590
10
1
0.1
0
5
10
15
Time (h)

(C)

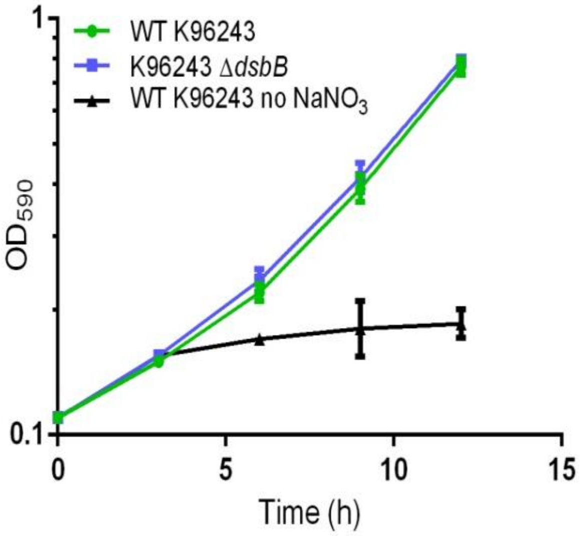

WT K96243
K96243 ΔdsbB
WT K96243 no NaNO3
OD590
1
0.1
0
5
10
15
Time (h)

Fig S1. Growth characteristics of *B. pseudomallei* strains grown aerobically (A, B) or (C) anaerobically. No difference in aerobic growth was observed between *B. pseudomallei* WT and Δ*dsbB* for strains K96243 or MSHR2511 (P 0.32 and P 0.67 respectively) (N=3). Growth of *B. pseudomallei* in the absence of oxygen not differ over the time course (P 0.18) (N=2) as determined by two way ANOVA. Error bars represent standard error of the mean.
